# Safety and Efficacy of Using Advanced Hybrid Closed Loop Off-Label in an Infant Diagnosed with Permanent Neonatal Diabetes Mellitus: A Case Report and a Look to the Future

**DOI:** 10.3390/children11101225

**Published:** 2024-10-09

**Authors:** Federico Pezzotta, Nicola Sarale, Giordano Spacco, Giacomo Tantari, Enrica Bertelli, Giulia Bracciolini, Andrea Secco, Giuseppe d’Annunzio, Mohamad Maghnie, Nicola Minuto, Marta Bassi

**Affiliations:** 1 Pediatric Clinic and Endocrinology Unit, IRCCS Istituto Giannina Gaslini, 16147 Genoa, Italy; dottorpezzottafederico@gmail.com (F.P.); martabassi@gaslini.org (M.B.); 2 Department of Neuroscience, Rehabilitation, Ophthalmology, Genetics, Maternal and Child Health, University of Genoa, 16126 Genoa, Italy; 3 Pediatric and Pediatric Emergency Unit, Children Hospital, Azienda Ospedaliera Universitaria SS Antonio e Biagio e C. Arrigo, 15121 Alessandria, Italy

**Keywords:** advanced hybrid closed loop (AHCL), continuous subcutaneous insulin infusion (CSII), neonatal diabetes mellitus (NDM), time in range (TIR), brain development

## Abstract

The case report shows the safety and efficacy of insulin treatment with Advanced Hybrid Closed Loop (AHCL) system in a young patient affected by permanent neonatal diabetes mellitus (PNDM) due to chromosome 8 deletion syndrome involving the GATA4 gene. In the first days of life, he presented hyperglycaemia and started an intravenous insulin infusion therapy, replaced by a continuous subcutaneous insulin infusion (CSII) with Medtronic Minimed 780G^®^ insulin pump (Medtronic, Northridge, CA, USA). At the age of 2 years, the off-label activation of SmartGuard^®^ automated insulin delivery mode led to a great improvement in glycaemic control, reaching all recommended targets. At the 1-month follow-up visit, Time in Range (TIR) increased from 66% to 79%, with a Time in Tight Range (TTIR) of 55% and a reduction of 11% in time in hyperglycaemia and of 2% in time in hypoglycaemia. During the entire follow-up, no episodes of ketoacidosis or severe hypoglycaemia were observed and the patient maintained the glycaemic recommended targets reached at 1 month. Maintaining optimal glycaemic control and reducing hyperglycaemia are essential for brain growth and neurocognitive development in young patients. AHCL use should be considered to ensure good glycaemic control in patients affected by neonatal diabetes.

## 1. Introduction

Neonatal Diabetes Mellitus (NDM) is a rare disease, with a reported incidence of 1 in 90,000 to 160,000 live births [1,2]. The defining feature of NDM is the onset of persistent hyperglycaemia within the first six months of life, resulting from impaired insulin function, which can include the missing or disturbed development of the pancreas, reduced pancreatic B-cell mass, disturbed B-cell function, or early islet cell destruction. This is mainly caused by a mutation in a single gene affecting pancreatic beta cell function. In a minority of cases, it is associated with other syndromes [3,4].

NDM can be classified as transient or permanent based on clinical evolution. The most common cause of transient neonatal diabetes mellitus (TNDM) is the overexpression of genes involved in insulin regulation, often due to an imprinting defect of a specific region of chromosome 6 (6q24) [5], while permanent neonatal diabetes mellitus (PNDM) is most commonly caused by mutations in genes that play a crucial role in the development and function of pancreatic beta cells. The most frequently mutated genes in PNDM are KCNJ11 and ABCC8 [6].

As recommended in the latest ISPAD guidelines, genetic testing is recommended in patients diagnosed with NDM under the age of 6 months, with the aim of identifying the genetic aetiology, which could subsequently influence the treatment of NDM. Patients carrying different mutations require different treatment, which may include insulin, oral antidiabetic agents, or even, rarely, bone marrow transplantation [7].

Currently, the most advanced insulin therapy systems in paediatric patients with type 1 diabetes mellitus (T1DM) are the Advanced Hybrid Closed Loop systems (AHCL) that automatically regulate insulin delivery according to glycaemic values, integrated with real-time continuous glucose monitoring (rt-CGM).

Minimed 780G is an insulin pump associated with rt-CGM and with a Proportional Integral Derivative (PID) algorithm. This system can deliver insulin in two different modes: the AHCL automatic mode (SmartGuard Mode^®^, Medtronic, Northridge, CA, USA), approved for use in children with T1DM above the age of 7 years and a total daily insulin dose (TDD) of at least 8 U/day, and the manual mode, which delivers user-programmed insulin maintaining only a predictive low-glucose suspend (PLGS) function, able to automatically suspend insulin delivery in case of predicted hypoglycaemia [8].

The treatment of patients with NDM with AHCL systems presents a number of challenges, the first of which is the unwieldiness of insulin therapy. This is particularly problematic in infants and children under two years of age, due to the variable number of daily meals, the type of food consumed, and the rapid change in energy consumption (and therefore in carbohydrate and insulin requirements) as the child grows.

This report describes the case of a small-for-gestational-age (SGA) late-preterm infant (34 + 6 weeks gestational age) diagnosed with NDM due to chromosome 8 deletion syndrome involving the GATA4 gene, which is associated with exocrine pancreatic insufficiency and congenital heart disease (partial atrioventricular canal defect—CAVD—with pulmonary valve stenosis) [9]. He was initially treated at the Neonatal Intensive Care Unit of the Hospital of Alessandria, before being transferred to the Neonatal and Paediatric Intensive Care Unit and the Paediatric Clinic and Endocrinology of the IRCCS Giannina Gaslini Institute, Genoa, Italy.

He was initially treated with intravenous insulin infusion and then shifted to continuous subcutaneous insulin infusion (CSII). The insulin pump used was Minimed 780G (Medtronic^®^), first in manual mode and, from the age of two years, in Advanced Hybrid Closed Loop (AHCL) automatic mode.

As recommended by the most recent guidelines, we referred to glycaemic control throughout CGM parameters: Time in Range (TIR, 70–180 mg/dL), Time In Tight Range (TTIR, 70–140 mg/dL), Time Below Range (TBR, 54–70 mg/dL), TBR < 54 mg/dL, Time Above Range (TAR, 180–250 mg/dL) and TAR > 250 mg/dL. The recommended targets for good glycaemic control are as follows: TIR > 70%, TBR 54–70 mg/dL < 4%, TBR < 54 mg/dL < 1%, TAR 180–250 mg/dL < 25%, TAR > 250 mg/dL < 5% [8,10,11]. The parents signed informed consent for the publication of this case report.

## 2. Case Report

The patient was born late preterm at a gestational age of 34 + 6 weeks. At birth, due to severe intra-uterine growth restriction (IUGR), he was small for gestational age (SGA) (1390 g, 1st percentile according to InesCharts—http://www.inescharts.com—URL accessed on 16 September 2024). The APGAR score was 7 at the 1st minute, rising to 8 at the 5th minute. Due to the newborn’s partial instability at birth, ventilatory support with continuous positive airway pressure (CPAP) and then with bilevel positive airway pressure (BPAP) was indicated. This was discontinued after 24 h due to the improvement of the newborn’s general conditions. Due to mild perinatal distress, an initial neurological assessment was performed by neonatal cranial ultrasound, which was normal.

Due to the presence of a systolic murmur in the first hours of life, a cardiac echocardiogram was performed, which showed partial CAVD associated with pulmonary stenosis, with a dysplastic pulmonary valve and a pulmonary artery pressure gradient of 80 mmHg. The presence of aorto-pulmonary collaterals was also noted.

At birth, the patient presented hypoglycaemia (35 mg/dL) and was treated with 10% glucose solution infusion, with a gradual improvement of glycaemic values. Six hours later, hyperglycaemia (250 mg/dL) was observed and antibiotic therapy with ampicillin and gentamicin was started on suspicion of sepsis; a 5% glucose solution infusion (at a rate of 2.5 mg/kg/min) was continued.

Based on these findings, continuous glucose monitoring was started and confirmed particularly high glucose levels, indicating the need for insulin administration, which was started intravenously with Humulin R insulin at a dose of 0.01 U/kg/h. On the seventh day of life, due to the normalisation of glycaemic values, an attempt of insulin withdrawal was made. The attempt failed (severe hyperglycaemia after 36 h) and insulin therapy was restarted.

Furthermore, poor growth with steatorrhea was observed in the first days of life. This condition was investigated by abdominal ultrasound and faecal elastase measurement, which confirmed the diagnosis of exocrine pancreatic insufficiency. Meal replacement therapy with pancreatic enzymes (Pancrelipasi) was started. Although the initial screening test for galactosemia was negative, subsequent tests were positive, leading to a lactose-free milk diet. Additionally, mixed hyperbilirubinemia with hypocholic stools and steatorrhea was observed, with no evidence of biliary dilatation on ultrasound.

In the first days of life, a blood transfusion was administered to restore low haemoglobin levels; on suspicion of thiamine-responsive megaloblastic anaemia syndrome (TRMA, characterised by megaloblastic anaemia, diabetes mellitus, and sensorineural deafness) [12,13,14], a therapeutic attempt with intramuscular thiamine hydrochloride at 25 mg/day was made. This treatment was subsequently discontinued after 7 days due to lack of benefit.

The set of these findings suggested a syndromic condition. Subsequently, genetic testing including Array-CGH, was conducted and revealed a deletion of chromosome 8 involving the GATA4 gene, which is associated with a condition characterised by neonatal diabetes, congenital heart disease, and pancreatic hypoplasia.

The patient was then transferred to the IRCCS Giannina Gaslini Institute for a pulmonary valvuloplasty, which was performed without significant complications. A brain MRI revealed a condition consistent with the deposition of ketone bodies or lactates, potentially resulting from hyperglycaemia or anaerobic glycolysis. Glucose monitoring was continued during hospitalisation, showing glycaemic values persistently above 200–400 mg/dL.

**Figure 1:**
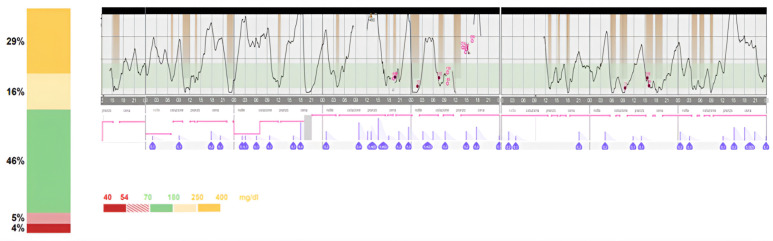
CGM parameters of the first 14 days of pump use. The glycaemic trend in the first few days after insulin pump placement shows numerous episodes of hyper- and hypoglycaemia, a sign of the difficulty in achieving a good glycaemic trend in a two-month-old patient.

**Figure 2:**
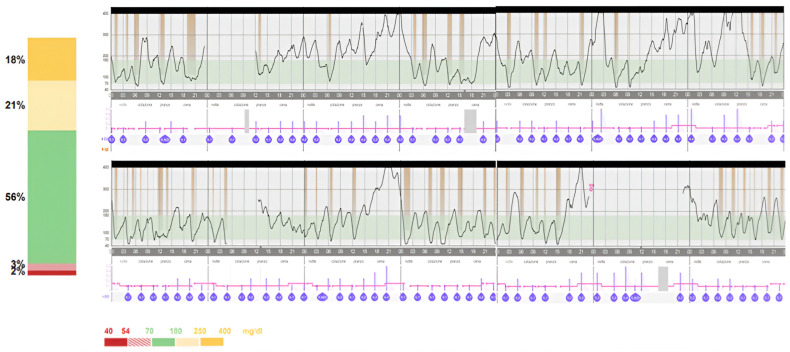
Glycaemic control 1 month after the placement of the insulin pump. Even with persistent hyperglycaemia due to partial meal control, glycaemic trends one month after insulin pump placement show an improvement, with a 10% increase in TIR.

In order to improve glycaemic control, intravenous insulin administration was substituted with a subcutaneous infusion through an insulin pump (Minimed 780G, Medtronic^®^). The pump was set with a basal insulin infusion rate of 0.025 U/h during the night and 0.075 U/kg/h during the day (TDD of 1.45 U). Initially, no boluses were planned for meals, and only the eventual correction suggested by the pump according to the glycaemic value was delivered at mealtimes. This choice was related to the fact that the patient consumed 8 meals/day of 60 mL of artificial formula (casein hydroxylate). Observing recurrent post-prandial hyperglycaemia (Figure 1), after 3 days, we decided to start meal boluses of 0.2 U, leading to an improvement of TIR of 10% and a reduction in TBR < 70 mg/dL of 4% (Figure 2). The patient was discharged with instructions for proper glycaemic and insulin pump management.

**Figure 3:**
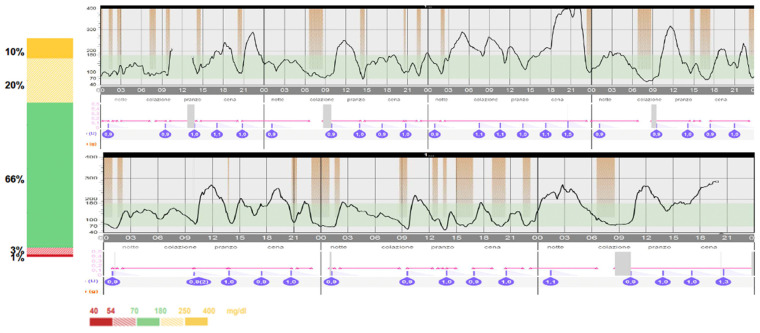
Glycaemic control 14 days before the activation of SmartGuard. The automatic mode was activated for our patient from the age of two to try to further improve glycaemic control.

**Figure 4:**
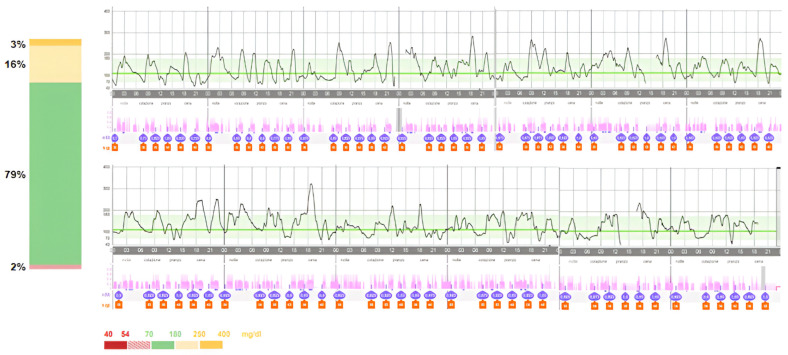
Glycaemic control 1 month after the activation of SmartGuard, showing the achievement of recommended glycaemic control with a TIR of 79% without increase in TBR.

The patient continued regular follow-up visits at the Diabetes Centre of our Institute, maintaining good and stable glycaemic control over time. In light of the demonstrated safety and effectiveness of SmartGuard Mode^®^ (Medtronic, Northridge, CA, USA) in T1DM patients below 7 years of age [15,16,17] and to further improve glycaemic control, when the patient reached a TDD of 8 units/die and at the age of 2 years, the SmartGuard mode was activated. Potential benefits and risks were explained to the family and written informed consent for the use of AHCL automatic mode off-label for age was obtained. The SmartGuard activation rapidly lead to a further improvement in glycaemic control, reaching recommended glycaemic targets defining good glycaemic control with a TIR of 79% and a TTIR of 55%, without increase in hypoglycaemia (TBR 2%). Figure 3 and Figure 4 show a comparison of glycaemic control 2 weeks before and 1 month after SmartGuard mode activation.

Since the activation of SmartGuard, the patient has been followed up regularly, consistently showing satisfactory glycaemic control as recommended by guidelines [8]. During the last follow-up visit, which occurred 6 months after the activation of the SmartGuard on the insulin pump, TIR was 72% and TTIR was 48%. No episodes of severe hypoglycaemia or DKA were reported during the entire period of use of the AHCL system.

Furthermore, during the last visit, weight, body growth, and psychomotor development were normal for age. The last encephalic MRI (performed at the age of 2 years and 4 months) showed the disappearance of the encephalic accumulation of ketone bodies, highlighting a slight non-specific thinning of the periventricular white substance.

## 3. Discussion

NDM is a heterogeneous disease that can be divided into two main types: transient and permanent. Each of these types has different causes and treatments, and, in most cases, is caused by a genetic mutation. TNDM typically resolves within the first few weeks or months of life. The most recurrent alteration causing TNDM (an imprinting defect in a specific region of chromosome 6, 6q24) leads to the overexpression of genes involved in insulin production and secretion. The initial treatment for TNDM is insulin therapy, which is used to control blood glucose levels. As TNDM often resolves spontaneously, insulin therapy can be discontinued as soon as the child’s pancreas restores its function. Furthermore, these patients may respond to several oral antidiabetic agents [18]. Nevertheless, due to the possible risk of recurrence, glycaemic values should be monitored over life [3,5].

In contrast, PNDM is a lifelong condition that may involve multiple gene mutations that alter the structure and/or function of the pancreatic beta cells responsible for insulin production. Lifelong insulin therapy has been the standard treatment for PNDM [18]. However, the identification of genetic causes, in particular, the two main mutations that cause PNDM (KCNJ11 and ABCC8), has led to the development of effective treatment options for many children with this type of diabetes, particularly those with KCNJ11 mutations. These include sulfonylurea drugs, which have been shown to be highly effective in stimulating insulin production in the pancreas, binding SUR1 subunits and inducing ATP-independent channel closure in response to high glucose, resulting in insulin secretion [19]. The transition from insulin injections to an oral therapy has been demonstrated to markedly enhance quality of life and glycaemic control.

Considering the high prevalence of genetic mutations underlying the diagnosis of NDM, genetic diagnosis is imperative for selecting the optimal treatment plan, as recommended by the latest ISPAD guidelines [7]. For patients with NDM carrying mutations in other less common genes, a different treatment approach is necessary. This typically involves insulin therapy, including the use of insulin pumps, which can be beneficial [7,20].

During the differential diagnosis, sepsis was initially suspected, but not confirmed due to the persistence of hyperglycaemia in the absence of other characteristic signs. Prior to the definitive genetic diagnosis, the presence of other clinical conditions in addition to hyperglycaemia and the need for insulin—such as poor growth and steatorrhea—had prompted the consideration of pancreatic insufficiency or agenesis, associated with cardiological involvement, given the finding of a heart murmur. This had led us to consider a more complex syndrome rather than an isolated neonatal diabetes, which is typical of other, more common mutations than the one found in our patient.

Once the GATA4 gene mutation was diagnosed, we realised that no other treatment options were possible in this case, such as switching to sulfonylureas, which has shown benefits in forms of neonatal diabetes characterised by mutations in the transport protein, especially in the most prevalent NDM-causing mutations (KCNJ11 and ABCC8) [21,22,23]. Nevertheless, the substitution of insulin therapy with this pharmacological agent has yet to be evaluated or approved for the treatment of NDM caused by less common genetic mutations. The options for insulin therapy were therefore limited to multiple-daily injection (MDI) therapy, traditional insulin pumps, and advanced pumps.

The potential use of MDI therapy has not been explored, given the influence of several factors, including the patient’s age, the complexity of administration, the possibility of patient discomfort, and the challenge of determining an appropriate dosage. Moreover, there is a paucity of research describing the treatment of NDM with MDI. In a case study by Lee et al. [24], a patient with NDM was treated with MDI for one week before switching to an AHCL system and finally to sulfonylurea therapy. During the initial week of MDI therapy, glycaemic control was challenging, with a TIR of 4%.

To the best of our knowledge, to date, there are only few data on the safety and efficacy of using insulin pumps in the treatment of NDM, for example, only case reports or series. A recent paper by Delvecchio et al. [5] described a case of TNDM treated with the MiniMed 780G system, which was used in manual mode because the total daily insulin dose was lower than the minimum dose required to activate the SmartGuard mode. Manual mode was found to be effective and safe, leading to a progressive reduction in TDD until suspension without evidence of hypoglycaemic episodes. Similar results were found in a previous review that analysed 19 cases of NDM treated with continuous subcutaneous insulin infusion (CSII), using either lispro (*n* = 9) or another insulin (*n* = 10) [25]. Lee et al. presented a case report of a young child with TNDM due to a pathologic mutation in the beta cell potassium ATP channel gene KCNJ11 treated with an AHCL system (Tandem Control-IQ) before the transition to sulfonylurea therapy. The glycaemic control of the patient improved, when compared to multiple-day injection therapy, reaching a TIR of 23%, without time spent in hypoglycaemia. During cross-titration with oral sulfonylurea, glycemia continued to improve (TIR 58%) and finally, on glyburide monotherapy, glycaemic control reached the recommended target (TIR 86%) [24]. Conversely, a paper by London et al. showed a case of non-identical twins presenting with NDM treated with CSII (MiniMed 640G), who manifested fluctuating blood glucose levels and frequent episodes of hypoglycaemia and hyperglycaemia at early follow-up; however, at 3 years of age, their glycated haemoglobin levels (6.5% and 7%, respectively) showed good glucometabolic control [26].

Currently, the use of Minimed 780G systems in SmartGuard mode is only approved for children over 7 years of age with a minimum TDD of 8 units. Several recent studies demonstrated the safety and efficacy of Minimed 780G systems in patients with T1DM younger than 7 years, even in children with a TDD < 8 U/day. Rapini et al. [27] conducted a two-centre prospective study enrolling 19 patients with T1DM between 2 and 7 years of age who were switched to AHCL from multiple daily injections or open-loop insulin therapy; after 6 months of follow-up, there was a significant reduction in median HbA1c (56.3 vs. 55 mmol/mol) and a reduction in mild hyperglycaemia (TAR 180–250 mg/dL, 25% vs. 21%) and in severe hyperglycaemia (TAR > 250 mg/dL, 11% vs. 7%), with a small increase in mild hypoglycaemia (TBR 54–70 mg/dL, 2% vs. 2.5%). TIR significantly increased compared with baseline (60% vs. 70%).

These results are consistent with the previous findings of Pulkkinen et al., who conducted a prospective study on 35 T1DM patients aged 2–7 years, evaluating the efficacy and the impact on parental diabetes distress of MiniMed 780G™ AHCL in SmartGuard™ mode used for 12 weeks. A significant increase in TIR of 8.3% was observed, without an increase in time in hypoglycaemia. Furthermore, a reduction in parental diabetes distress was observed through a significant reduction score of specific questionnaires after 12-week use [15].

In another pilot study with 11 children aged 2–6 years by Abraham et al. [28], in which a 2-week phase in manual mode was followed by a 6-week AHCL phase, the mean TIR was 58% at enrolment and 64.9% in manual mode; TIR increased to 72.6% in AHCL, meeting the recommended target. Time spent in hypoglycaemia also remained within the clinical recommended targets.

Tornese et al. [29] conducted a retrospective analysis of data concerning 12 children <7 years of age with T1DM who were on the Medtronic MiniMed 780G system with SmartGuard features for at least 6 months. A significant increase in TIR (65.5% vs. 57%) and a significant decrease in TAR (>180 mg/dL, 33% vs. 39%) and TBR (<70 mg/dL, 2.5% vs. 4%) were observed at the beginning of SmartGuard mode; differences in TAR and TIR also remained at 6 and 9 months but not at 3 months. No episodes of severe hypoglycaemia were recorded. Moreover, in this study, the minimum reported daily dose was 4 units; even when the TDD was <8 units, the system was constantly running in SmartGuard mode.

A substantial body of evidence, primarily from ISPAD guidelines, indicates that the earliest possible initiation of AHCL therapy following a diagnosis of diabetes is crucial to ensure optimal glycaemic control. This is of particular importance in younger patients, where difficulties in meal management (which become more frequent and unpredictable at preschool age, depending on the child’s behaviour) may lead to a worsening of TIR [7,20].

This is crucial for patients diagnosed with either precocious T1DM or NDM, who experience a higher risk of potential suboptimal cognitive and motor development, especially due to ketoacidosis at onset, hypoglycaemic episodes, and prolonged time in hyperglycaemia [30,31,32]. The brain of infants and young children is highly sensitive to metabolic disturbances. Potential abnormalities, particularly in white matter, have been identified in several neuroimaging studies of young brains exposed to glycaemic extremes, as seen in T1DM and NDM [33,34,35].

The precise mechanisms by which early brain development is affected by T1DM or NDM remain unclear. Metabolic conditions such as hyperglycaemia and ketoacidosis at diagnosis may make the brain more vulnerable to subsequent metabolic insults [36,37,38]. Furthermore, these neurocognitive and psychomotor consequences may not become apparent until later in childhood. It can be reasonably assumed that optimal glycaemic control will give young children with diabetes the best chance to concentrate, participate, and learn in preschool and school.

To the best of our knowledge, this is the first case report of a newborn with PNDM treated with the MiniMed^®^780G on SmartGuard mode (AHCL algorithm). Consistently with previous studies conducted on young patients with T1DM, a clinically significant improvement in glycaemic control was also observed in this very young patient affected by NDM. After only 1 month of activation of the AHCL system, an increase in TIR of 13% (66% vs. 79%) and a reduction in time in hyperglycaemia of 11% (30% vs. 19%) were observed, without an increase in time in hypoglycaemia. A TTIR of 55%, higher than the 50% recommended, was reached [39]. Therefore, the use of SmartGuard allowed the young patient to gain more than 3 h per day of time in euglycemia per day and to reduce the time of exposure to hyperglycaemia by 2.5 h per day. Minimed 780G was shown to be effective and safe in this patient, since no episodes of severe hypoglycaemia or ketoacidosis were reported during the entire follow-up period.

Reaching the recommended glycaemic targets, which is extremely difficult in very young children, not only represents a therapeutic success and a contribution to the improvement of family diabetes distress, but it has recently been proven to be essential to ensure the best possible neurocognitive development of young patients. White matter growth is reduced, and white matter microstructure is altered in the brains of young children with diabetes, due in part to the adverse effects of hyperglycaemia, and may contribute to mild cognitive deficits in this population [35]. The last brain MRI performed on our patient showed a slight non-specific thinning of the periventricular white substance. During follow-up, it will be essential to monitor the patient’s neurocognitive development and the evolution of the brain MRI, to evaluate the impact of the early use of AHCL on the cognitive development and on the brain structure.

The role of the diabetes team is essential during follow-up to periodically review the correct diabetes management and the use of the insulin pump, to maintain over time the optimal glycaemic control reached [40].

In conclusion, the use of the AHCL system in a young patient with NDM showed excellent results in terms of efficacy and safety, and this therapeutic option should be considered for infants with NDM.

## Data Availability

Restrictions apply to the availability of these data. Data were obtained from an online database (Carelink^®^) and are available from the database with the permission of Carelink^®^ and the patient’s parents.
